# Supplementary material for: Stakeholder perspectives on interventions to improve HIV pre-exposure prophylaxis uptake and continuation in Lesotho: A participant-ranked preferences study
Source: PLOS Glob Public Health. 2023 Sep 27;3(9):e0001423. doi: 10.1371/journal.pgph.0001423 (PMC10529554; doi:10.1371/journal.pgph.0001423)
Supplement: S1 Table — (PDF) [file pgph.0001423.s002.pdf]

Table S1: Barrier and intervention candidates presented to participants during the card sorting and ranking exercise

A. Barriers for PrEP Uptake	B. Interventions for PrEP Uptake
A1. Limited awareness of PrEP<br>A2. Difficulty in communicating with health providers about sexual matters<br>A3. Difficulty in accessing PrEP<br>A4. Fear of HIV testing<br>A5. Perceived stigma<br>A6. Risk perception<br>A7. Perception that PrEP is not effective<br>A8. Limited decision making power<br>A9. Concern of side effects<br>A10. Lack of social support	B1. Community-based HIV testing<br>B2. Workplace HIV testing and PrEP promotion<br>B3. PrEP promotion in *Shebeens* (local bars)<br>B4. Facility-based PrEP Promotion Package<br>B5. Mass media campaign<br>B6. HIV testing and PrEP promotion in partnership with faith-based organizations and religious leaders<br>B7. Partner with traditional healers for HIV testing and PrEP Promotion<br>B8. Partner with Community Based Organizations for HIV testing and PrEP Promotion<br>B9. Partner with community leaders for HIV testing and PrEP Promotion
**C. Barriers for PrEP Retention**	**D. Interventions for PrEP Retention**
C1. Perceived and/or experienced stigma<br>C2. Risk perception<br>C3. Perception that PrEP is not effective<br>C4. Decision making power<br>C5. Side effects<br>C6. Medication regimen<br>C7. Lack of social support<br>C8. Factors of daily life	D1. Home/community PrEP delivery<br>D2. Increase PrEP prescription quantity<br>D3. SMS reminders<br>D4. Telephone calls<br>D5. Extended health facility hours<br>D6. Intensive counseling<br>D7. PrEP administration e.g. injection or implant<br>D8. Peer counseling<br>D9. Incentives<br>D10. Support groups
