# Supplementary material for: Stakeholder perspectives on interventions to improve HIV pre-exposure prophylaxis uptake and continuation in Lesotho: A participant-ranked preferences study
Source: PLOS Glob Public Health. 2023 Sep 27;3(9):e0001423. doi: 10.1371/journal.pgph.0001423 (PMC10529554; doi:10.1371/journal.pgph.0001423)
Supplement: S1 Text — (PDF) [file pgph.0001423.s003.pdf]

UPTAKE AND ADHERENCE: CARD SORTING AND RANKING EXERCISE

As we come to the end of our discussion, I would like to engage you in a short sorting and ranking exercise. I have a list of things that we have heard make it difficult for people to start and keep using PrEP. I also have a list of possible interventions (solutions) that could be used to overcome those challenges. In this activity:

- *Sorting* means that you group barriers/interventions into categories that I give you; and
- *Ranking* means that you arrange the barriers/interviews in order based on importance.

1.0 PrEP INITIATION AND UPTAKE

In this first section, I will be asking you to sort and rank barriers (challenges) and interventions (solutions) you think make it difficult for men and women in Lesotho to ***start*** taking PrEP.

1.1 Barriers to PrEP Initiation

These are some reasons we have heard make it difficult for people to ***start*** taking PrEP.

Interviewer: *Show laminated cards with barriers to respondent, and read each one to them out loud*

1. **Awareness**: little awareness of PrEP as a tool for HIV prevention
2. **Difficulty in/concern of communicating** with health providers about sexual matters
3. Difficulty in **accessing PrEP**
4. Fear of **HIV testing**
5. Perceived **stigma**. E.g. being on PrEP is a sign of being HIV positive
6. **Risk perception**: individual does not see themselves as being at high risk for HIV infection
7. Perception that **PrEP is not effective** in preventing HIV infection
8. **Decision making** power: Individual feels like they do not have the power to make the decision to begin using PrEP
9. **Side effects**: concern about possible long-term and/or short-term side effects
10. Lack of **social support**: family, partner, or community is not supportive of the individual taking PrEP. Or concern that use of PrEP can cost them a relationship with their family members, romantic partners etc.

1.1.1 Barriers to PrEP Initiation for *WOMEN*

a. Pile sorting

Based on your personal opinions and experience with PrEP, I would like to ask you to ***sort*** the barriers that make it difficult for ***WOMEN*** in Lesotho to start taking PrEP into the following piles (categories):

A= Biggest barriers
B= Somewhat of a barrier
C= Not a barrier at all

Remember, there are no right or wrong answers. Also, note that the piles do not have to have the same number of barriers.

> ***Interviewer***:
> - *As the respondent is sorting the barriers, ask them their reasoning for deciding to categorize the barriers in the way they did.*
> - *List down how the respondent categorized each barrier under the* ***WOMEN*** *column in table 1 below. NOTE: the order in which you list the barriers in each pile is not important.*

b. Ranking

Using only the options from the "biggest barriers" category above, arrange those barriers in such a way that **1=biggest barrier**. Remember, there is no right or wrong order, just place the cards in order as you see fit. Please note that no 2 responses can have the same ranking. You should the barriers are in ascending order with no tied ranking.

> ***Interviewer***:
> - *As the respondent is ranking the barriers, ask them their reasoning for deciding to naming [insert barrier ranked #1 here] as the biggest barrier for* ***WOMEN;*** and the reasoning for the other barriers.
> - *List down the ranking of each barrier under the* ***WOMEN*** *column in table 2 below.*

1.1.2 Barriers to PrEP Initiation for *MEN*

a. Pile sorting

I would like to ask you to sort the same barriers. This time, however, think about the barriers that make it difficult for ***MEN*** in Lesotho to ***start*** taking PrEP into the following piles (categories):

A= Biggest barriers
B= Somewhat of a barrier
C= Not a barrier at all

Remember, there are no right or wrong answers. Also, note that the piles do not have to have the same number of barriers.

> ***Interviewer***:
> - *As the respondent is sorting the barriers, ask them their reasoning for deciding to categorize the barriers in the way they did.*
> - *List down how the respondent categorized each barrier under the* ***MEN*** *column in table 1 below. NOTE: the order in which you list the barriers in each pile is not important.*

b. Ranking

Using only the options from the "biggest barriers" category above, arrange those barriers in such a way that **1=biggest barrier**. Remember, there is no right or wrong order, just place the cards in order as you see fit. Please note that no 2 responses can have the same ranking. You should the barriers are in ascending order with no tied ranking.

Interviewer:

- *As the respondent is ranking the barriers, ask them their reasoning for deciding to naming [insert barrier ranked #1 here] as the biggest barrier for* ***MEN;*** and the reasoning for the other barriers.
- *List down the ranking of each barrier under the* ***MEN*** *column in table 2 below.*

Table 1: Pile sorting of barriers for PrEP initiation

	A. Biggest barriers	B. Somewhat of a barrier	C. Not a barrier at all
**Women**	[___],[___],[___],[___],[___] [___],[___],[___],[___],[___]	[___],[___],[___],[___],[___] [___],[___],[___],[___],[___]	[___],[___],[___],[___],[___] [___],[___],[___],[___],[___]
**Men**	[___],[___],[___],[___],[___] [___],[___],[___],[___],[___]	[___],[___],[___],[___],[___] [___],[___],[___],[___],[___]	[___],[___],[___],[___],[___] [___],[___],[___],[___],[___]
*Total responses	[___]	[___]	[___]
*Note: total responses should add up to the total number of choices			

Table 2: Ranking of biggest barriers for PrEP initiation

**Sorting categories**		**Target population**	
	**Ranking**	**Women**	**Men**
***Biggest barriers**	1=biggest barrier		
	2		
	3		
	4		
	5		
	6		
	7		
	8		
	9		
	10		
*Note: the number of items ranked here should add up to the total number of items under the "biggest barriers" category			

1.2 Interventions to Improve PrEP Initiation

Now, we will discuss some interventions (solutions) you think may be helpful in overcoming challenges related to starting PrEP. Below are some interventions (solutions) you think may be helpful in overcoming challenges related to starting PrEP

Interviewer: *Show laminated cards with barriers to respondent, and read each one to them out loud*

1. **Community-based HIV testing** through community health workers or mobile vans with subsequent PrEP promotion and counseling for those testing negative
2. **Workplace HIV testing** and PrEP promotion
3. PrEP promotion (flyers, posters etc.) through **village/city Shebeens**
4. **Facility-based PrEP Promotion Package** (fliers, posters etc)
5. **Mass media campaign** (social media, radio, TV, newspapers, etc.)
6. HIV testing and PrEP promotion in partnership with **faith-based organizations and religious leaders**
7. HIV testing and PrEP promotion in partnership with **traditional healers**
8. HIV testing and PrEP promotion in partnership with **grassroots community organizations**
9. HIV testing and PrEP promotion in partnership with **community leaders**

1.2.1 Interventions to improve Uptake PrEP initiation for ***WOMEN***

a. Pile sorting

Based on your personal opinions and experience with PrEP, I would like to ask you to ***sort*** the interventions that would encourage ***WOMEN*** in Lesotho to get tested for HIV and start taking PrEP f eligible. Sort the interventions into the following piles (categories):

A= Most helpful interventions
B= Somewhat helpful interventions
C= Not helpful at all

Remember, there are no right or wrong answers. Also, note that the piles do not have to have the same number of barriers.

Interviewer:

- *As the respondent is sorting the interventions, ask them their reasoning for deciding to categorize the interventions in the way they did.*
- *List down how the respondent categorized each intervention under the* ***WOMEN*** *column in table 3 below. NOTE: the order in which you list the barriers in each pile is not important.*

b. Ranking

Now I would like you to pretend that I am giving you M100,000 to spend on solutions that will encourage people to get tested for HIV and to start using PrEP if eligible. Pretend you are a leader and have to spend money on the following interventions (solutions). Using only the interventions from the "most helpful" category above, arrange the interventions in such a way that **1=invest most money**.

Remember, there is no right or wrong order, just place the cards in order as you see fit. Please note that no 2 responses can have the same ranking. You should the barriers are in ascending order with no tied ranking.

> ***Interviewer****:*
> - *As the respondent is ranking the intervention, ask them their reasoning for deciding to naming [insert intervention ranked #1 here] as the one that should revive the most money for* ***WOMEN;*** and the reasoning for the order of other interventions.
> - *List down the ranking of each intervention under the* ***WOMEN*** *column in table 4 below.*

1.2.2 Interventions to improve PrEP initiation for *MEN*

a. Pile sorting

I would like to ask you to think about the same interventions. This time, however, think about the interventions that would be best suited for ***MEN*** in Lesotho.

Based on your personal opinions and experience with PrEP, I would like to ask you to ***sort*** the interventions that would encourage ***MEN*** in Lesotho to get tested for HIV and start taking PrEP if eligible. Sort the interventions into the following piles (categories):

A= Most helpful interventions
B= Somewhat helpful interventions
C= Not helpful at all

Remember, there are no right or wrong answers. Also, note that the piles do not have to have the same number of barriers.

> ***Interviewer****:*
> - *As the respondent is sorting the interventions, ask them their reasoning for deciding to categorize the interventions in the way they did.*
> - *List down how the respondent categorized each intervention under the* ***MEN*** *column in table 3 below. NOTE: the order in which you list the barriers in each pile is not important.*

b. Ranking

Now I would like you to pretend that I am giving you M100,000 to spend on solutions that will encourage people to get tested for HIV and to start using PrEP if eligible. Pretend you are a leader and have to spend money on the following interventions (solutions). Using only the interventions from the "most helpful" category above, arrange the interventions in such a way that **1=invest most money**.

Remember, there is no right or wrong order, just place the cards in order as you see fit. Please note that no 2 responses can have the same ranking. You should the barriers are in ascending order with no tied ranking.

> ***Interviewer****:*
> - *As the respondent is ranking the intervention, ask them their reasoning for deciding to naming [insert intervention ranked #1 here] as the one that should revive the most money for* ***MEN;*** and the reasoning for the order of other interventions.
> - *List down the ranking of each intervention under the* ***MEN*** *column in table 4 below.*

Table 3: Pile sorting of interventions to improve Uptake of HIV Testing and PrEP initiation

	**A. Most helpful**	**B. Somewhat helpful**	**C. Not helpful**
**Women**	[___],[___],[___],[___],[___]<br>[___],[___],[___],[___],[___]	[___],[___],[___],[___],[___]<br>[___],[___],[___],[___],[___]	[___],[___],[___],[___],[___]<br>[___],[___],[___],[___],[___]
**Men**	[___],[___],[___],[___],[___]<br>[___],[___],[___],[___],[___]	[___],[___],[___],[___],[___]<br>[___],[___],[___],[___],[___]	[___],[___],[___],[___],[___]<br>[___],[___],[___],[___],[___]
*Total responses	[___]	[___]	[___]
*Note: total responses should add up to the total number of choices			

Table 4: Ranking of "most helpful" interventions to improve Uptake of HIV Testing and PrEP initiation

**Sorting categories**		**Target population**	
	**Ranking**	**Women**	**Men**
***Most helpful interventions**	**1=Invest most money**		
	2		
	3		
	4		
	5		
	6		
	7		
	8		
	9		
*Note: the number of items ranked here should add up to the total number of items under the "most helpful interventions" category			

2.0 PrEP ADHERENCE

In this second section, we will be sorting and ranking barriers (challenges) you think make it difficult for men and women in Lesotho to ***keep*** taking PrEP, once they have started. We will also discuss some interventions (solutions) you think may be helpful in overcoming challenges related to adhering to PrEP.

2.1 Barriers to PrEP Adherence

Below are some reasons we have heard make it difficult for people to ***keep*** taking PrEP, after they have started.

Interviewer: *Show laminated cards with barriers to respondent, and read each one to them out loud*

1. Perceived and/or experienced **stigma**. E.g. being on PrEP is a sign of being HIV positive
2. **Risk perception**: individual does not see themselves as being at high risk for HIV infection
3. **Knowledg**e on PrEP: Perception that PrEP is not effective in preventing HIV infection
4. **Decision making** power: Individual feels like they do not have the power to make the decision to stay on PrEP
5. **Side effects**: concern about possible long-term and short-term side effects
6. **Medication regimen**: the act of taking a pill every single day is demanding
7. Lack of **social support**: family, partner, or community is not supportive of the individual taking PrEP. Or concerned that use of PrEP can cost them a relationship with their family members, romantic partners etc.
8. Factors of **daily life**: run out of medication; forgot because of influence of alcohol; individual away from home etc.

2.1.1 Barriers to PrEP Adherence for *Women*

a. Pile sorting

Based on your personal opinions and experience with PrEP, I would like to ask you to ***sort*** the barriers that make it difficult for ***WOMEN*** in Lesotho to ***keep*** taking PrEP, after they have started, into the following piles (categories):

A= Biggest barriers
B= Somewhat of a barrier
C= Not a barrier at all

Remember, there are no right or wrong answers. Also, note that the piles do not have to have the same number of barriers.

Interviewer*:*<br>• *As the respondent is sorting the barriers, ask them their reasoning for deciding to categorize the barriers in the way they did.*<br>• *List down how the respondent categorized each barrier under the* ***WOMEN*** *column in table 5 below. NOTE: the order in which you list the barriers in each pile is not important.*

b. Ranking

Using only the options from the "biggest barriers" category above, arrange those barriers in such a way that **1=biggest barrier**. Remember, there is no right or wrong order, just place the cards in order as you see fit. Please note that no 2 responses can have the same ranking. You should the barriers are in ascending order with no tied ranking.

Interviewer**:*<br>• *As the respondent is ranking the barriers, ask them their reasoning for deciding to naming [insert barrier ranked #1 here] as the biggest barrier for **WOMEN; and the reasoning for the other barriers.<br>• *List down the ranking of each barrier under the **WOMEN** column in table 6 below.*

2.1.2 Barriers to PrEP Adherence for *MEN*

a. Pile sorting

I would like to ask you to sort the same barriers. This time, however, think about the barriers that make it difficult for ***MEN*** in Lesotho to ***keep*** taking PrEP, after they have started into the following piles (categories):

A= Biggest barriers
B= Somewhat of a barrier
C= Not a barrier at all

Remember, there are no right or wrong answers. Also, note that the piles do not have to have the same number of barriers.

***Interviewer**:*<br>• *As the respondent is sorting the barriers, ask them their reasoning for deciding to categorize the barriers in the way they did.*<br>• *List down how the respondent categorized each barrier under the **MEN** column in table 1 below. NOTE: the order in which you list the barriers in each pile is not important.*

c. Ranking

Using only the options from the "biggest barriers" category above, arrange those barriers in such a way that **1=biggest barrier**. Remember, there is no right or wrong order, just place the cards in order as you see fit. Please note that no 2 responses can have the same ranking. You should the barriers are in ascending order with no tied ranking.

Interviewer**:*<br>• *As the respondent is ranking the barriers, ask them their reasoning for deciding to naming [insert barrier ranked #1 here] as the biggest barrier for **MEN; and the reasoning for the other barriers.<br>• *List down the ranking of each barrier under the **MEN** column in table 2 below.*

Table 5: Pile sorting of barriers for PrEP Adherence

	A. Biggest barriers	B. Somewhat of a barrier	C. Not a barrier at all
**Women**	[___],[___],[___],[___],[___]<br>[___],[___],[___],[___],[___]	[___],[___],[___],[___],[___]<br>[___],[___],[___],[___],[___]	[___],[___],[___],[___],[___]<br>[___],[___],[___],[___],[___]
**Men**	[___],[___],[___],[___],[___]<br>[___],[___],[___],[___],[___]	[___],[___],[___],[___],[___]<br>[___],[___],[___],[___],[___]	[___],[___],[___],[___],[___]<br>[___],[___],[___],[___],[___]
*Total responses	[___]	[___]	[___]
*Note: total responses should add up to the total number of choices			

Table 6: Ranking of biggest barriers for PrEP adherence

**Sorting categories**		**Target population**	
	**Ranking**	**Women**	**Men**
***Biggest barriers**	1=biggest barrier		
	2		
	3		
	4		
	5		
	6		
	7		
	8		
	9		
	10		
*Note: the number of items ranked here should add up to the total number of items under the "biggest barriers" category			

2.2 Interventions to Improve PrEP Adherence

Now, we will discuss some interventions (solutions) you think may be helpful in overcoming challenges related to adhering to PrEP. Below are some interventions (solutions) you think may be helpful in overcoming challenges related to starting PrEP

Interviewer: *Show laminated cards with barriers to respondent, and read each one to them out loud*

1. **Home/community delivery** of PrEP pills through CHWs once clients have initiated PrEP
2. **Pill prescription quantity**: Prescribe more pills to reduce frequency of pick up
3. **SMS reminders**: send text messages every day to PrEP clients for them to take the pill
4. **Telephone calls**: have regular telephone calls to remind PrEP clients to take their pills
5. **Extended health facility hours**: open the health facility later so that PrEP clients can pick up medications at later times
6. **Counseling**: provide more in-depth counseling on the importance to adhering to PrEP from health care providers
7. **Pill delivery**: have PrEP administered in a different way e.g. injection or implant

8. **Peer counseling**: have others on PrEP counsel individuals new to PrEP
9. **Incentives**: offer financial or non-financial rewards for adherence on PrEP
10. **Support groups**: form groups for PrEP users to encourage adherence

2.2.1 Interventions to improve PrEP initiation for *WOMEN*

a. Pile sorting

Based on your personal opinions and experience with PrEP, I would like to ask you to ***sort*** the interventions that would encourage ***WOMEN*** in Lesotho to get keep taking PrEP once they have started. Sort the interventions into the following piles (categories):

A= Most helpful interventions
B= Somewhat helpful interventions
C= Not helpful at all

Remember, there are no right or wrong answers. Also, note that the piles do not have to have the same number of barriers.

> ***Interviewer****:*
> - *As the respondent is sorting the interventions, ask them their reasoning for deciding to categorize the interventions in the way they did.*
> - *List down how the respondent categorized each intervention under the* ***WOMEN*** *column in table 7 below. NOTE: the order in which you list the barriers in each pile is not important.*

b. Ranking

Now I would like you to pretend that I am giving you M100,000 to spend on solutions that will encourage people to get tested for HIV and to start using PrEP if eligible. Pretend you are a leader and have to spend money on the following interventions (solutions). Using only the interventions from the "most helpful" category above, arrange the interventions in such a way that **1=invest most money**.

Remember, there is no right or wrong order, just place the cards in order as you see fit. Please note that no 2 responses can have the same ranking. You should the barriers are in ascending order with no tied ranking.

> ***Interviewer****:*
> - *As the respondent is ranking the intervention, ask them their reasoning for deciding to naming [insert intervention ranked #1 here] as the one that should revive the most money for* ***WOMEN;*** and the reasoning for the order of other interventions.
> - *List down the ranking of each intervention under the* ***WOMEN*** *column in table 8 below.*

2.2.2 Interventions to improve PrEP initiation for <u>*MEN*</u>

a. Pile sorting

I would like to ask you to think about the same interventions. This time, however, think about the interventions that would be best suited for <u>***MEN***</u> in Lesotho.

Based on your personal opinions and experience with PrEP, I would like to ask you to <u>***sort***</u> the interventions that would encourage <u>***MEN***</u> in Lesotho to continue taking PrEP once they start. Sort the interventions into the following piles (categories):

A= Most helpful interventions
B= Somewhat helpful interventions
C= Not helpful at all

Remember, there are no right or wrong answers. Also, note that the <u>piles do not have to have the same number of barriers</u>.

> ***Interviewer***:
> - *As the respondent is sorting the interventions, ask them their reasoning for deciding to categorize the interventions in the way they did.*
> - *List down how the respondent categorized each intervention under the* <u>***MEN***</u> *column in table 7 below. NOTE: the order in which you list the barriers in each pile is not important.*

b. Ranking

Now I would like you to pretend that I am giving you M100,000 to spend on solutions that will encourage people to keep taking PrEP once they have started. Pretend you are a leader and have to spend money on the following interventions (solutions). Using only the interventions from the "most helpful" category above, arrange the interventions in such a way that **1=invest most money**.

Remember, there is no right or wrong order, just place the cards in order as you see fit. Please note that <u>no 2 responses can have the same ranking</u>. You should the barriers are in ascending order with no tied ranking.

> ***Interviewer***:
> - *As the respondent is ranking the intervention, ask them their reasoning for deciding to naming [insert intervention ranked #1 here] as the one that should revive the most money for* <u>***MEN;***</u> and the reasoning for the order of other interventions.
> - *List down the ranking of each intervention under the* <u>***MEN***</u> *column in table 8 below.*

Table 7: Pile sorting of interventions to improve PrEP adherence

	A. Most helpful	B. Somewhat helpful	C. Not helpful
**Women**	[___],[___],[___],[___],[___] [___],[___],[___],[___],[___]	[___],[___],[___],[___],[___] [___],[___],[___],[___],[___]	[___],[___],[___],[___],[___] [___],[___],[___],[___],[___]
**Men**	[___],[___],[___],[___],[___] [___],[___],[___],[___],[___]	[___],[___],[___],[___],[___] [___],[___],[___],[___],[___]	[___],[___],[___],[___],[___] [___],[___],[___],[___],[___]
*Total responses	[___]	[___]	[___]
*Note: total responses should add up to the total number of choices			

Table 8: Ranking of "most helpful" interventions to improve PrEP adherence

**Sorting categories**		**Target population**	
	**Ranking**	**Women**	**Men**
***Most helpful interventions**	**1=Invest most money**		
	2		
	3		
	4		
	5		
	6		
	7		
	8		
	9		
*Note: the number of items ranked here should add up to the total number of items under the "most helpful interventions" category			
