# Supplementary material for: Stakeholder perspectives on interventions to improve HIV pre-exposure prophylaxis uptake and continuation in Lesotho: A participant-ranked preferences study
Source: PLOS Glob Public Health. 2023 Sep 27;3(9):e0001423. doi: 10.1371/journal.pgph.0001423 (PMC10529554; doi:10.1371/journal.pgph.0001423)
Supplement: Attachment — Submitted filename: Response to reviewers_29June2023.docx [file pgph.0001423.s004.docx]

June 29, 2023

Dear Dr. Hlongwa,

We would like to thank you and the reviewers for taking the time to provide helpful and constructive feedback on our manuscript titled: “*Stakeholder perspectives on interventions to improve HIV pre-exposure prophylaxis uptake and continuation in Lesotho: A participant-ranked preferences study*”. Specifically, we are grateful to reviewer 1 who notes that “[the] methodology employed is novel and makes an important contribution to understanding not only the scope of perceived barriers to PrEP initiation and persistence, but also the relative importance of such barriers and how they vary by gender and among different stakeholders”. We also thank the second reviewer who mentioned, “the manuscript is well written and easy to follow”. The input offered through the peer review process has undoubtedly made our manuscript stronger.

Below, please see our point-by-point response to the comments received. We believe our revisions (also highlighted via track changes in the attached manuscript) have responded to the recommendations by you and the reviewers.

Additionally, kindly note slight changes in the authorship order. This change, agreed upon by all authors, was done to reflect contributions made by authors more accurately.

Sincerely,

The Authors

Joy J. Chebet

Shannon A. McMahon

Rachel P. Chase

Tapiwa Tarumbiswa

Chivimbiso Maponga

Esther Mandara

Till Bärnighausen

Pascal Geldsetzer

**Point-by-point responses to reviewers**

| **Comments** | **Response** |
| --- | --- |
| **Editor/Journal** | |
| We have noticed that you have uploaded Supporting Information files, but you have not included a list of legends. Please add a full list of legends for your Supporting Information files after the references list | We have added this list of legends at the end of the document, after the references |
| In the online submission form, you indicated that "A deidentified version of our dataset will be made available in a publicly accessible data repository upon acceptance of the manuscript for publication". All PLOS journals now require all data underlying the findings described in their manuscript to be freely available to other researchers, either 1. In a public repository, 2. Within the manuscript itself, or 3. Uploaded as supplementary information. This policy applies to all data except where public deposition would breach compliance with the protocol approved by your research ethics board. If your data cannot be made publicly available for ethical or legal reasons (e.g., public availability would compromise patient privacy), please explain your reasons by return email and your exemption request will be escalated to the editor for approval. Your exemption request will be handled independently and will not hold up the peer review process, but will need to be resolved should your manuscript be accepted for publication. One of the Editorial team will then be in touch if there are any issues. | This deidentified dataset is now available and has been uploaded as supplementary material (S3) |
| **Reviewer 1** | |
| This study employed a novel staged sorting and ranking method for identifying barriers and interventions to PrEP uptake and persistence among multi-level stakeholders in the context of national PrEP implementation in Lesotho. The methodology employed is novel and makes an important contribution to understanding not only the scope of perceived barriers to PrEP initiation and persistence, but also the relative importance of such barriers and how they vary by gender and among different stakeholders. Ranking methods are advantageous because they force participants to choose relative importance, though they can be unreliable due to the high cognitive load of ordering long lists of items. The authors address this limitation by first asking participants to identify the most important items, thereby shortening the list of items for consideration before ranking. The manuscript makes an important contribution towards the literature, and particularly toward identifying strategies of PrEP delivery that are tailored towards the needs and preferences of different populations, and I think it merits publication once the authors address the issues I outline below. My main suggestion is that the authors provide greater detail and justification for the barriers and interventions chosen, along with proposed mechanisms for how interventions are hypothesized to address the included barriers. Please see my detailed comments below: | We thank the reviewer for this positive feedback and their note on the significance of this paper in the field. Comments raised by reviewer have been addressed below. |
| The authors do not provide sufficient information on how the barriers and interventions were selected for the survey. Please provide additional theoretical justification for why these particular barriers and interventions were chosen, including a theoretical model if one was used. | Following the reviewer’s comments, we have included more detail on how barriers and interventions presented to participants were selected. This part of the methods section now reads:  *Barriers and interventions included in this study were sourced a priori from literature [11–17] and through discussions with collaborators in the PrEP field. Following the ecological model, we selected barriers to represent all levels of the health system – individual, interpersonal, societal, contextual and structural factors. The barriers and interventions to PrEP implementation that were presented to participants in this study are shown in Table S1 in the appendix* |
| The study nicely highlights important barriers and important interventions for PrEP uptake and persistence as perceived by multi-level stakeholders. However, I think more could be done to connect identified barriers to interventions of greatest importance. For example, stigma, factors of daily life, and medication regimen were selected as barriers to persistence for men, and multi-month prescriptions and intensive counseling were selected as most important interventions, though it is likely that each of these interventions is addressing different barriers. Use of a theoretical model or mechanism map (#1 above) would help elucidate hypothesized relationships, and an analysis stratified by most important barrier reported would provide further empirical evidence for relationships between barriers and selected interventions. For example, it would be helpful to know if the Smith set of most important interventions differed for individuals who selected stigma vs. factors of daily life as the greatest barrier to PrEP persistence | While the reviewer highlights an interesting approach to connect the barriers and interventions of greatest importance, the study was not designed with this in mind. We, instead, sought to identify barriers and interventions that are of high priority to stakeholders, and can therefore be studied in greater depth in future studies. This has now been clarified in the methods and discussion sections of the manuscript.  Methods:  *the present study sought to understand stakeholder perspectives on which barriers to PrEP uptake and interventions encourage its use with the ultimate aim of identifying salient interventions for further investigation in future.*  Discussion:  *As PrEP is adopted more globally, it is essential to understand end-user preferences while also being cognizant of practicalities policymakers, implementing partners and health providers navigate. While literature proposes interventions to improve PrEP coverage, it is impractical to study the effectiveness of all interventions in resource-limited settings. Therefore, obtaining and ranking the views of stakeholders allows for the most promising interventions to be distilled.* |
| Can the authors share the materials used to describe the barriers and interventions to participants? Some of the barriers are difficult to interpret without additional detail on how they were presented (e.g. “medication regimen”, “factors of daily life”). | The instrument used in the study has now been added as supplemental material S2 |
| My understanding is that gender-stratified results are based on the entire sample reporting importance of barriers and interventions for men and women separately. The authors note that men are under-represented in the study, however I would suggest expanding on this limitation to mention that the results for men are mostly based on perceptions of barriers and importance as reported by women. Alternatively, the authors could consider a sensitivity analysis that was stratified by participant gender (i.e. men reporting on barriers/interventions for men, and women reporting on barriers/interventions for women). If results are similar, this would strengthen the validity of the findings. | Given that our sample was majority female, we did not analyze results based on the sex of participants. Rather, we asked the participants what they thought were the biggest barrier for men and women in Lesotho. This is clarified in the methods section, where we note:  *To gain gender-specific insight, each participant (regardless of their gender) was asked to sort and rank each set of barriers and interventions for men and women separately* |
| The sorting/ranking method used is novel and makes an important contribution. In the discussion, I would suggest adding a brief paragraph describing how this method differs from other ranking methods (e.g. best-worst scaling, Q-methodology), including advantages and disadvantages. | Based on the reviewer’s suggestion, we have added the following statements in the methods and discussion sections of the manuscript:  Methods:  *The use of social choice theory, and the Smith set/Condorcet method specifically, allowed us to reach group consensus and identify robust interventions to be considered to improve PrEP coverage [31,32]. These techniques allow for nuanced participant-centered insights on priority barriers/interventions and integrates varied perspectives from PrEP stakeholders involved in implementation and use of PrEP. Given the broad nature of our inquiry, other preference elicitation methods, such as discrete choice experiments, were inappropriate [33].*  Discussion:  *This study employed a novel approach to analyze ranked data by applying social choice theory to health. Traditionally, preference data has been gathered qualitatively, with quantitative methods (including discrete choice experiments) being employed in recent years. However, these studies have focused mainly on PrEP formulation preferences [42–44]. We chose to use the Smith voting analysis methods since, unlike political elections, more than one salient candidate is acceptable.* |
| Intervention, Line 108: the references for PrEP implementation studies in sub-Saharan Africa are quite dated (2016) and there are more recent studies published in this area. Please update references and place this study into context with more recent studies. | These references have now been updated to more recent studies published in the preceding 3 years |
| In table 2, the demographics are presented for 105 healthcare workers who participated in a larger study, but only 51 participated in the present study. Please include demographic data specific to the 51 participants in the present study. | Data specific to the 51 health providers included in this study were prematurely anonymized. To explain this, we have included the following statement in the results section:  *Note that while 105 health providers were included in the larger implementation study, 51 of them participated in the card sorting and ranking exercise. However, data from this subset were prematurely anonymized. Therefore, health provider demographic information from the total sample of 105 health providers is used to approximate this study’s health provider sample* |
| In table 3, I think there is an error in the ‘Policy makers/implementing partners’ column for Barriers to PrEP uptake. Should limited awareness of PreP and perceived stigma be indicated for women instead of men? | We thank the reviewer for pointing this out. This correction has been made to table 3. |
| **Reviewer 2** |  |
| In this manuscript, the authors present descriptive findings from a ranked preferences exercise among a range of HIV pre-exposure prophylaxis (PrEP) implementation stakeholders (including program staff, healthcare workers and end users of PrEP), designed to understand perceptions of barriers and potential interventions to: 1) PrEP uptake and 2) retention in PrEP programs, among men and women in Lesotho. Overall, the manuscript is well written and easy to follow. The results are descriptive without statistical analyses. Novel aspects of the study include consideration of stakeholder perceptions as ranked preferences determined with a card sorting exercise and inclusion of persons who are currently on PrEP, no longer on PrEP but PrEP experienced, and those who declined PrEP. The key weaknesses of the manuscript are as follows. | We are grateful to the reviewer for their feedback and have addressed the comments raised below. |
| First, the authors note in the Introduction that, “At present, little evidence on interventions to achieve high PrEP coverage in the general population is available, particularly in sub-Saharan Africa.” The authors have overlooked several publications from the SEARCH study in East Africa in which PrEP has been offered to a general population (for example, please see Koss et al, “Uptake, engagement, and adherence to pre-exposure prophylaxis offered after population HIV testing in rural Kenya and Uganda: 72-week interim analysis of observational data from the SEARCH study,” Lancet HIV, 2020, PMID: 32087152), as well as the Partners Demonstration project that incorporated PrEP into HIV care clinics (reaching both men and women, see reference: Irungu et al, "Integration of pre-exposure prophylaxis services into public HIV care clinics in Kenya: a pragmatic stepped-wedge randomised trial," Lancet Global Health, 2021, PMID: 34798031). | The reviewer raised a good point. More recent studies have been added to the background section. The section highlighted by the reviewer now reads:  *While recent studies have contributed evidence on specific interventions to improve PrEP uptake more broadly – such as counseling [25] and integration of PrEP services into HIV clinics [26]– more data on other promising interventions to achieve high PrEP coverage in the general adult population are needed, particularly in the sub-Saharan Africa region.* |
| Second, the authors do not justify why they used the card sorting exercise to determine ranked preferences as opposed to other quantitative preference elicitation methods, such as best worst sampling surveys, or discrete choice experiments. The authors should provide a brief explanation at how they arrived at this method. For an overview of types of preference elicitation methods, see Soekhai et al, “Methods for exploring and eliciting patient preferences in the medical product lifecycle: a literature review,” Drug Discov Today, 2019, PMID: 31077814; as well as Kerkhoff et al, “A world of choices: preference elicitation methods for improving the delivery and uptake of HIV prevention and treatment,” Curr Opin HIV AIDS. 2023, PMID: 36409315. | Based on the reviewer’s suggestion, we have added the following statements in the methods and discussion sections of the manuscript:  Methods:  *The use of social choice theory, and the Smith set/Condorcet method specifically, allowed us to reach group consensus and identify robust interventions to be considered to improve PrEP coverage [31,32]. These techniques allow for nuanced participant-centered insights on priority barriers/interventions and integrates varied perspectives from PrEP stakeholders involved in implementation and use of PrEP. Given the broad nature of our inquiry, other preference elicitation methods, such as discrete choice experiments, were inappropriate [33].*  Discussion:  *This study employed a novel approach to analyze ranked data by applying social choice theory to health. Traditionally, preference data has been gathered qualitatively, with quantitative methods (including discrete choice experiments) being employed in recent years. However, these studies have focused mainly on PrEP formulation preferences [42–44]. We chose to use the Smith voting analysis methods since, unlike political elections, more than one salient candidate is acceptable* |
| Third, the authors recently published findings from qualitative interviews with (what appears to be) the same population of stakeholders in PLoS Global Public Health (reference: Geldsetzer et al, “Knowledge and attitudes about HIV pre-exposure prophylaxis: Evidence from in-depth interviews and focus group discussions with policy makers, healthcare providers, and end-users in Lesotho,” 2022, PMID: 36962565). A weakness of the present manuscript (under review) is a lack of discussion of what the card sorting exercise adds to the prior publication, and it seems unusual that the authors have not cited this manuscript as a useful reference for readers to get a better picture of the viewpoints of this group of stakeholders in Lesotho. Did the qualitative interviews and analysis inform the card sorting exercise, or vice versa? | We have clarified that this card sorting and ranking exercise was conducted as part of a larger study. Additionally, we have cited our previously published article discussing findings from another sub-study. The study design section of the manuscript now reads:  *Data were collected between March and April 2019 as part of a larger implementation research study on stakeholder perspectives on the Lesotho PrEP program and scale-up [30]. While other sub-studies aimed to explore participant knowledge of PrEP and motivations for use, the present study sought to understand stakeholder perspectives on which barriers to PrEP uptake and interventions encourage its use with the ultimate aim of identifying salient interventions for further investigation in future.* |
